# Targeting endothelin receptors A and B attenuates the inflammatory response and improves locomotor function following spinal cord injury in mice

**DOI:** 10.3892/ijmm.2014.1751

**Published:** 2014-04-22

**Authors:** JIAN GUO, YIQIAO LI, ZHENNIAN HE, BIN ZHANG, YONGHUAN LI, JIANGHUA HU, MINGYUAN HAN, YUANLIN XU, YONGFU LI, JIE GU, BO DAI, ZHONG CHEN

**Affiliations:** 1 Department of Orthopaedic Surgery, Ningbo Beilun People Hospital, Ningbo, Zhejiang 315800, P.R. China; 2 Central Laboratory, Ningbo Beilun People Hospital, Ningbo, Zhejiang 315800, P.R. China; 3 Department of Orthopaedic Surgery, The First Affiliated Hospital of College of Medicine, Zhejiang University, Hangzhou, Zhejiang 310000, P.R. China

**Keywords:** endothelin receptors, inflammatory response, long-term neurological improvement, oxidative stress

## Abstract

After spinal cord injury (SCI), the disruption of blood-spinal cord barrier by activation of the endothelin (ET) system is a critical event leading to leukocyte infiltration, inflammatory response and oxidative stress, contributing to neurological disability. In the present study, we showed that blockade of ET receptor A (ETAR) and/or ET receptor B (ETBR) prevented early inflammatory responses directly via the inhibition of neutrophil and monocyte diapedesis and inflammatory mediator production following traumatic SCI in mice. Long-term neurological improvement, based on a series of tests of locomotor performance, occurred only in the spinal cord-injured mice following blockade of ETAR and ETBR. We also examined the post-traumatic changes of the microenvironment within the injured spinal cord of mice following blockade of ET receptors. Oxidative stress reflects an imbalance between malondialdehyde and superoxide dismutase in spinal cord-injured mice treated with vehicle, whereas blockade of ETAR and ETBR reversed the oxidation state imbalance. In addition, hemeoxygenase-1, a protective protease involved in early SCI, was increased in spinal cord-injured mice following the blockade of ETAR and ETBR, or only ETBR. Matrix metalloproteinase-9, a tissue-destructive protease involved in early damage, was decreased in the injured spinal cord of mice following blockade of ETAR, ETBR or a combination thereof. The findings of the present study therefore suggested an association between ETAR and ETBR in regulating early pathogenesis of SCI and determining the outcomes of long-term neurological recovery.

## Introduction

The blood-spinal cord barrier (BSCB) is a highly specialized structural, transport and biochemical barrier within the central nervous system (CNS). Similar to the blood-brain barrier (BBB), the BSCB is primarily formed by endothelial cells interconnected by tight junctions, which limits passive diffusion of blood-borne solutes and actively transports nutrients into the spinal cord (1,2). BSCB dysfunction leading to early inflammatory response and oxidative stress contributes to secondary pathogenesis following traumatic spinal cord injury (SCI) (3–10). BSCB disruption by traumatic SCI also generates harmful substances, including endothelins (ETs) (11–15), matrix metalloproteinases (MMPs) (7,9,10), inflammatory cytokines and reactive oxygen species (ROS) (3–6,8) that can induce programmed neuronal death and permanently impair neuron function. This is exemplified by studies showing that the blockade of ET receptors or ET-converting enzyme (ECE) activity in brain endothelial cells and glia, which results in endothelial hyperpermeability and cerebral vasoconstriction, reduces leukocyte infiltration into the injured spinal cord, which is associated with significant recovery of motor and neurological functions following SCI (16–18). Therefore, the brain ET system is considered to be a therapeutic target of SCI.

The ET system consists of two G-protein-coupled receptors (ET receptors A and B, ETRs), three peptides (ET-1, ET-2 and ET-3), and two activating peptidases (ECE-1 and ECE-2). It is the most potent vasoconstrictor and is essential for embryonic development, vascular remodeling, and wound healing (19,20). Excessive activation of the ET system can be detrimental, leading to multidimensional pathological conditions, including BBB or BSCB disruption following ischemic brain injury and traumatic SCI, as well as inflammation (20,21). For example, the ET system is found throughout the brain as its components are synthesized in vascular, neuronal, and glial cells. Expression pattern of ET system components in many discrete brain areas suggests a variety of possible functions (19–22). ET-1 is the predominant neural ET and plays a critical role in abnormal vascular endothelial cell permeability and inflammation after SCI, while the upregulation of ET-1 modulates behavior and the metabolism without affecting cerebral blood flow (23). In addition, ETs exert their effects through the activation of ET receptor A (ETAR) and/or ET receptor B (ETBR) (19,22,23). In normal spinal cord, ETAR is found predominantly in vascular smooth muscle cells and primary afferent nerve fibers, whereas ETBR is abundantly expressed in endothelial cells, radial glia, a small population of astrocytes, and epithelial tissues (23,24). Following SCI, vascular ETAR/ETBR activation plays a critical role in post-traumatic ischemia, and astrocyte-only ETBR activation is associated with reactive gliosis (23–25). However, until recently, there was a lack of consensus regarding which ETR subtype was the key determinant of oxidative stress and functional recovery after SCI, and there has been controversy regarding the exact cellular targets of ETAR and ETBR in the injured spinal cord.

In the present study, we examined the effects of ETAR and/or ETBR blockade on early SCI pathogenesis and long-term neurological recovery in murine models. Our results demonstrated that ETR blockage markedly reduced inflammatory responses and oxidative stress, ameliorated MMP-9 activation, and enhanced long-term neurological function in SCI mice. The results confirm additive pathogenic roles for ETAR and ETBR in the injured spinal cord and may aid in the identification of a set of putative therapeutic targets for neural tissue damage after SCI.

## Materials and methods

### SCI

All the procedures were conducted in accordance with the National Institutes of Health Guide for the Care and Use of Laboratory Animals and with approval from the Animal Subjects Committee at Zhejiang University. Adult female C57BL/6 (18–22 g) mice were anesthetized with chloral hydrate (500 mg/kg) and subjected to a moderate spinal cord contusion injury. A laminectomy was performed at the T9 level, and a 2-g weight was dropped 5 cm onto the exposed dura mater. After SCI, the skin was closed with wound clips. Animal body temperature was maintained at 37°C with a warming blanket throughout the surgery and during the recovery from anesthesia (26). For the sham-operated controls (SHAM), the animals underwent a T9 laminectomy without contusion injury.

### Drug treatment

BQ123 and/or BQ788 (both from Sigma, St. Louis, MO, USA) dissolved in sterile phosphate-buffered saline (PBS) were administered to corresponding SHAM and SCI mice (SHAM + BQ123, SHAM + BQ788, SHAM + BQ123 + BQ788, SCI + BQ123, SCI + BQ788 and SCI + BQ123 + BQ788) via intraperitoneal injection (10 mg/kg, respectively) (27–29) at 1 day before SCI and then further treated once a day for 6 weeks for behavioral testing or for the indicated time-points (2, 4, 6 and 24 h and 4 days) for other experiments post-injury. PBS for vehicle control (VEH) was administered in corresponding SHAM and SCI mice (SHAM + VEH and SCI + VEH). Significant side effects resulting from BQ123 and/or BQ788 treatment, such as changes in body weight or an increase in mortality, were not observed during our experiments.

### Leukocyte infiltration assessment

To confirm the depletion of neutrophils, three blood smears, prepared 1 day post-injury were processed with a Hema 3^®^ stain kit (Fisher Scientific, Pittsburgh, PA, USA). At least 300 white blood cells were counted, and the percentage of neutrophils relative to the total number of white blood cells was determined. To confirm monocyte depletion, blood samples were taken at 4 days post-injury. A hematology automated white blood cell analyzer (HemaVet^®^ 850) was used to quantify monocytes. Data are presented as relative percentages relative to the total number of white blood cells.

### Quantitative polymerase chain reaction (qPCR)

Total RNA was prepared with the RNeasy kit (Qiagen). Complementary DNA synthesis and reverse transcriptase PCR were performed using a previously described method (30). The primers used were: tumor necrosis factor-α (TNF-α), forward, 5′-CCCAGA CCCTCACACTCAGAT-3′ and reverse, 5′-TTGTCCCTT GAGAGAACCTG-3′; interleukin-1β (IL-1β), forward, 5′-GCA GCTACCTATGTCTTGCCCGTG-3′ and reverse, 5′-GTCGTT GCTTGTCTCTCCTTGTA-3′; IL-6, forward, 5′-AAGTTT CTCTCCGCAAGATACTTCCAGCCA-3′ and reverse, 5′-AGGCAAATTTCCTGGTTATATCCAGTT-3′; inducible nitric oxide synthase (iNOS), forward, 5′-CTCCATGACTCT CAGCACAGAG-3′ and reverse, 5′-GCACCGAAGATATCC TCATGAT-3′.

### Enzyme-linked immunosorbent assay (ELISA)

To determine cytokine levels, the lesion site was rapidly dissected and homogenized in PBS at 24 h after SCI. After centrifugation at 4°C for 15 min at 900 × g, the supernatants were used to measure the concentrations of TNF-α, IL-1β and IL-6 (R&D Systems, Minneapolis, MN, USA), iNOS, malondialdehyde (MDA), and superoxide dismutase (SOD) (Cusabio Biotech Co., Ltd., Hubei, China) using corresponding ELISA kits.

### Western blot analysis

A 0.5-cm length of cord, centered over the site of impact and representing the epicenter, was lysed on ice for 30 min with 50 mM Tris-HCl (pH 7.5), 150 mM NaCl, 1 mM EDTA, 1 mM EGTA, 25 mM NaF, 5 mM sodium pyrophosphate, 1 mM Na_3_VO_4_, and protease inhibitors (Roche). Cell lysates were clarified by centrifugation at 12,000 × g for 15 min at 4°C, and the supernatants were collected and assayed for the protein concentration using a BCA protein assay kit (Pierce, Rockford, IL, USA). Total cell lysates were prepared, and western blot analysis was performed as described previously (30). The antibodies used were: anti-hemeoxygenase-1 (anti-HO-1, sc-10789; Santa Cruz Biotechnology, Inc., Santa Cruz, CA, USA), anti-MMP-9 (ab7299; Abcam), anti-α/β-tubulin (no. 2148; Cell Signaling Technology). Tubulin served as a loading control.

### Gelatin zymography

MMP-9 activity at 24-h post-injury was examined by gelatin zymography based on a previously described method (26). The epicenter of the injured spinal cord (0.5 cm in length) was homogenized in lysis buffer containing 50 mM Tris-HCl (pH 8.0), 150 mM NaCl, 1% NP-40, 0.5% deoxycholate, and 0.1% SDS. Then, 50-μg protein samples were loaded onto 8% SDS-polyacrylamide gels and copolymerized with gelatin (1 mg/ml; Sigma). After electrophoresis, renaturation was achieved by incubation of the gel in 2.5% Triton X-100 for 30 min and in substrate buffer (50 mM Tris-HCl at pH 8.5, 5 mM CaCl_2_) for 48 h at 37°C. The gel was stained with Coomassie blue solution for 4 h and then de-stained with 40% methanol/10% acetic acid. For quantitative analysis, gels were scanned, and the positive band was measured using NIH ImageJ software.

### Behavioral analysis

Three different behavioral tests were performed to evaluate functional improvements after SCI. The 9-point Basso Mouse Scale (BMS) was used to examine locomotor recovery in an open field (53×10^8^×5.5 cm) (31). This rating scale takes into account limb movement, stepping, coordination, and trunk stability. Mice were tested at 1 and 3 days and weekly thereafter until euthanasia at 6 weeks post-injury. Performance on a rotarod and the ability to traverse a wire grid were evaluated, in sequence, at 40, 41 and 42 days post-injury. Three experiments were conducted daily, with a total of nine trials for each test. In each of these tests, the average score from each mouse was used to calculate the mean.

### Statistical analysis

Statistical analysis was performed using GraphPad Prism (GraphPad Software, Inc., La Jolla, CA, USA). Statistical significance was defined at P<0.05. Data were presented as the mean ± standard error of the mean (SEM) of three independent experiments.

## Results

### Blockade of ETAR and ETBR reduced leukocyte infiltration in SCI mice

**Figure 1:**
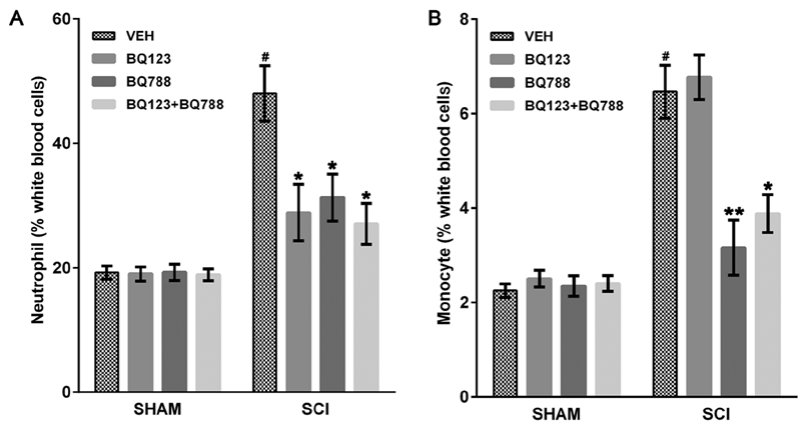
Treatment with BQ123 and/or BQ788 reduces neutrophils and monocytes, respectively, in the blood of spinal cord injury (SCI) mice. Data are the mean ± standard error of the mean (SEM) of three independent experiments. ^*^P<0.05, ^**^P<0.01, SCI mice treated with BQ123 and/or BQ788 vs. SCI + vehicle control (VEH) mice; ^#^P<0.01, SCI + VEH mice vs. SHAM mice (n=3/group). (A) The number of neutrophils in blood was manually counted in blood smears stained with the Hema 3^®^ Stain kit. (B) The number of monocytes in blood was quantified using an automated white blood cell analyzer (HemaVet).

As previously described (32), the early leukocyte influx contributes to secondary pathogenesis in the injured spinal cord. Therefore, we investigated the effects of ETR blockade on leukocyte influx by performing differential blood cell counts. Compared with each SHAM mouse, SCI + VEH mice showed an elevated number of neutrophils and monocytes (Fig. 1). The number of neutrophils in peripheral blood was reduced in SCI mice at 24 h after blockade of ETAR, ETBR or both (Fig. 1A). Blockade of ETBR or ETAR and ETBR reduced circulating monocytes 4 days after SCI (Fig. 1B). Notably, blockade of only ETAR did not affect the number of circulating monocytes in SCI mice (Fig. 1B).

### Blockade of ETAR and ETBR inhibited inflammatory mediator expression in SCI mice

**Figure 2:**
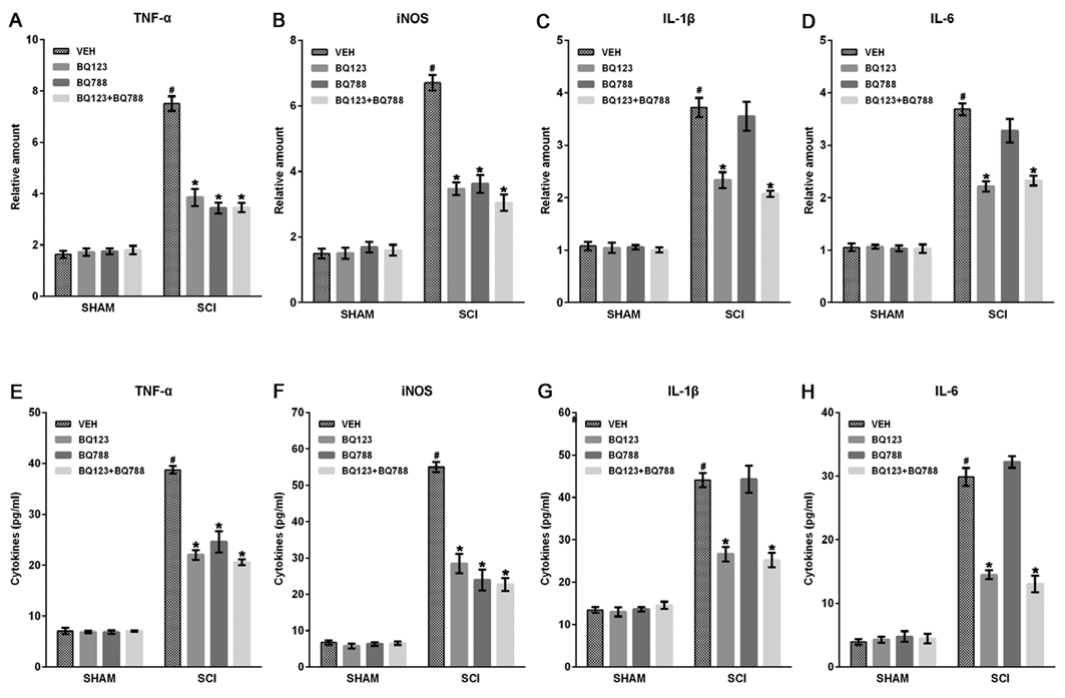
Treatment with BQ123 and/or BQ788 inhibits the expression of cytokines and inflammatory mediators in spinal cord injury (SCI) mice. Data represent mean ± standard error of the mean (SEM) of three independent experiments. ^*^P<0.05, SCI mice treated with BQ123 and/or BQ788 vs. SCI + vehicle control (VEH) mice; ^#^P<0.01, SCI + VEH mice vs. SHAM mice (n=3/group). (A–D) Quantitative analysis of reverse transcriptase PCR of tumor necrosis factor-α (TNF-α), interleukin-1β (IL-1β) (at 2 h), IL-6, inducible nitric oxide synthase (iNOS) (at 6 h) in SHAM and SCI mice left untreated or treated with BQ123 and/or BQ788 post-injury. (E–H) Enzyme-linked immunosorbent assay (ELISA) of TNF-α, IL-1β, IL-6 and iNOS at 24 h in SHAM and SCI mice left untreated or treated with BQ123 and/or BQ788 post-injury.

After SCI, leukocyte infiltration following BSCB disruption initiated inflammatory responses, leading to a secondary cascade of brain trauma due to the production of inflammatory mediators, such as TNF-α, IL-1β, IL-6 and iNOS (30). Therefore, we analyzed the effect of ETR blockade on the expression of inflammatory mediators by reverse-transcriptase PCR and ELISA assays at indicated time-points after SCI. Results showed that the mRNA expression levels of TNF-α, IL-1β (at 2 h), IL-6, and iNOS (at 6 h) were upregulated post-injury, and blockade of ETAR and ETBR significantly reduced the levels of these inflammatory mediators compared with VEHs (Fig. 2A–D). In addition, the blockade of only ETAR, but not ETBR, decreased IL-1β and IL-6 expression in spinal cords post-injury (Fig. 2B and C). ELISA assays also showed that the blockade of the two ETRs significantly inhibited the production of TNF-α, IL-1β, IL-6 and iNOS at 24 h after SCI (Fig. 2E–H).

### Reduction in oxidative stress in SCI mice following ETAR and ETBR blockade

**Figure 3:**
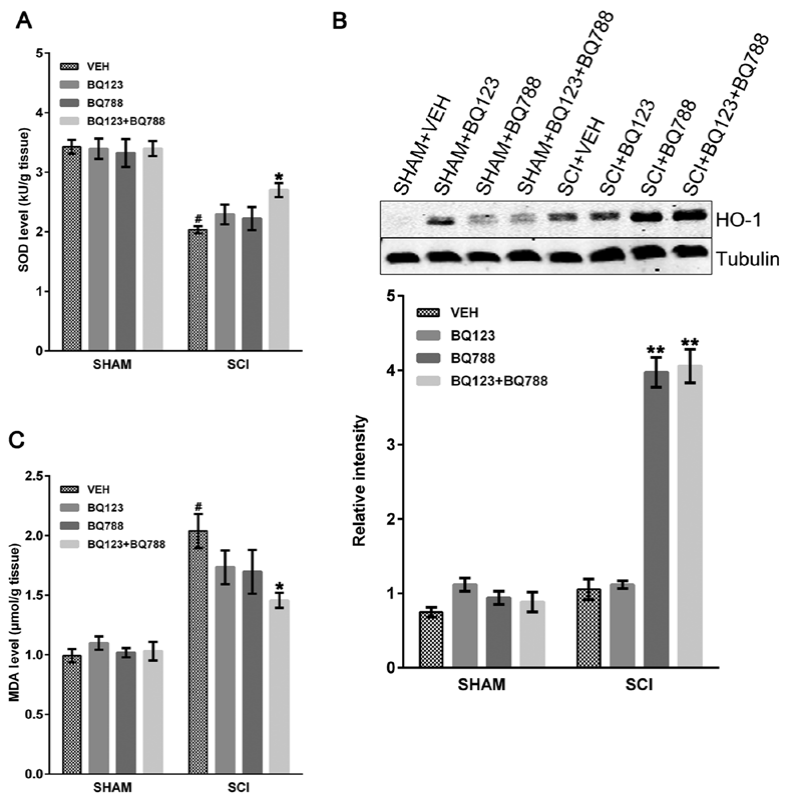
Treatment with BQ123 and/or BQ788 inhibits the expression of oxidative stress markers in spinal cord injury (SCI) mice. Data are presented as mean ± standard error of the mean (SEM) of three independent experiments. ^*^P<0.05, ^**^P<0.01, SCI mice treated with BQ123 and/or BQ788 vs. SCI + vehicle control (VEH) mice; ^#^P<0.01, SCI + VEH mice vs. SHAM mice (n=3/group). (A) Enzyme-linked immunosorbent assay (ELISA) of SOD at 24 h in SHAM and SCI mice left untreated or treated with BQ123 and/or BQ788 post-injury. (B) Quantitative analysis of western blot of HO-1 at 24 h in SHAM and SCI mice left untreated or treated with BQ123 and/or BQ788 post-injury. Data are presented as mean ± SEM of three independent experiments. (C) ELISA of MDA at 24 h in SHAM and SCI mice left untreated or treated with BQ123 and/or BQ788 post-injury. MDA, malondialdehyde.

Oxidative stress markers (HO-1, MDA and SOD) in injured mice with or without blockade of ETAR and ETBR were examined. SOD is an enzyme that neutralizes oxygen-free radicals and protects cells from being oxidized by superoxide toxicity (33). SOD is consumed during oxidative stress in a variety of pathological conditions. SCI caused a significant decrease in SOD level in SCI + VEH mice compared to SHAM mice, and the blockade of ETAR and ETBR significantly rescued the SOD level at 24 h after SCI (Fig. 3A). However, the blockade of only ETAR or ETBR did not rescue the SOD level in SCI mice (Fig. 3A). Similarly, HO-1, the inducible form of HO, is an important defense mechanism against early oxidative stress (5,34). Immunoblot assays revealed a greater increase in HO-1 levels at 24 h in SCI mice with blockade of ETBR or both ETAR and ETBR compared to SCI + VEH mice (Fig. 3B). By contrast, no differences were observed between SCI mice with only ETAR blockade and SCI + VEH mice (Fig. 3B). Nonetheless, levels of MDA, the final product of lipid peroxidation (33), were increased at 24 h after SCI and reduced in SCI mice with ETAR and ETBR blockade, whereas no statistically significant differences were found in SCI mice with blockade of only ETAR or ETBR (Fig. 3C).

### Blockade of ETAR and ETBR reduced MMP-9 expression following SCI

**Figure 4:**
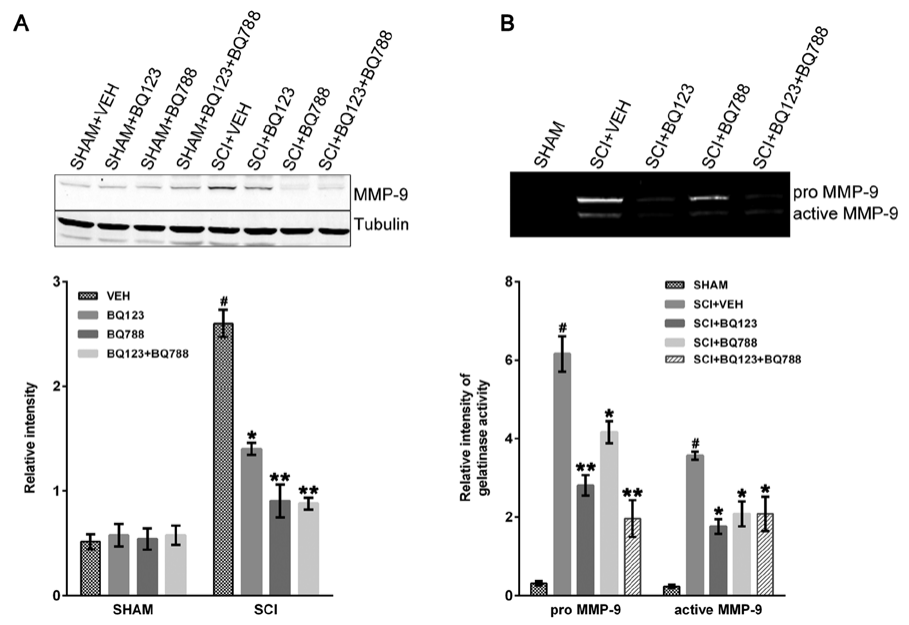
Treatment with BQ123 and/or BQ788 inhibits the expression of matrix metalloproteinase-9 (MMP-9) in spinal cord injury (SCI) mice. Data are the mean ± standard error of the mean (SEM) of three independent experiments. ^*^P<0.05, ^**^P<0.01, SCI mice treated with BQ123 and/or BQ788 vs. SCI + vehicle control (VEH) mice; ^#^P<0.01, SCI + VEH mice vs. SHAM mice (n=3/group). (A) Quantitative analysis of western blot of MMP-9 at 24 h in SHAM and SCI mice left untreated or treated with BQ123 and/or BQ788 post-injury. (B) Quantitative analysis of gelatin zymography of MMP-9 at 24 h in SHAM and SCI mice left untreated or treated with BQ123 and/or BQ788 post-injury.

MMP-9 plays a critical role in early neutrophil infiltration and long-term functional impairment after SCI (26). The immunoblot results revealed that MMP-9 was markedly increased at 24 h in SCI + VEH mice compared to control and was reduced in SCI mice with blockade of ETAR, ETBR, or a combination thereof (Fig. 4A).

The pro- and active forms of MMP-9 were evaluated in SHAM and SCI mice by gelatin zymography. The two forms were significantly reduced in SCI mice that were depleted of ETAR, ETBR, or both ETRs 24-h post-injury (Fig. 4B).

### Blockade of ETAR and ETBR improved functional recovery after SCI

**Figure 5:**
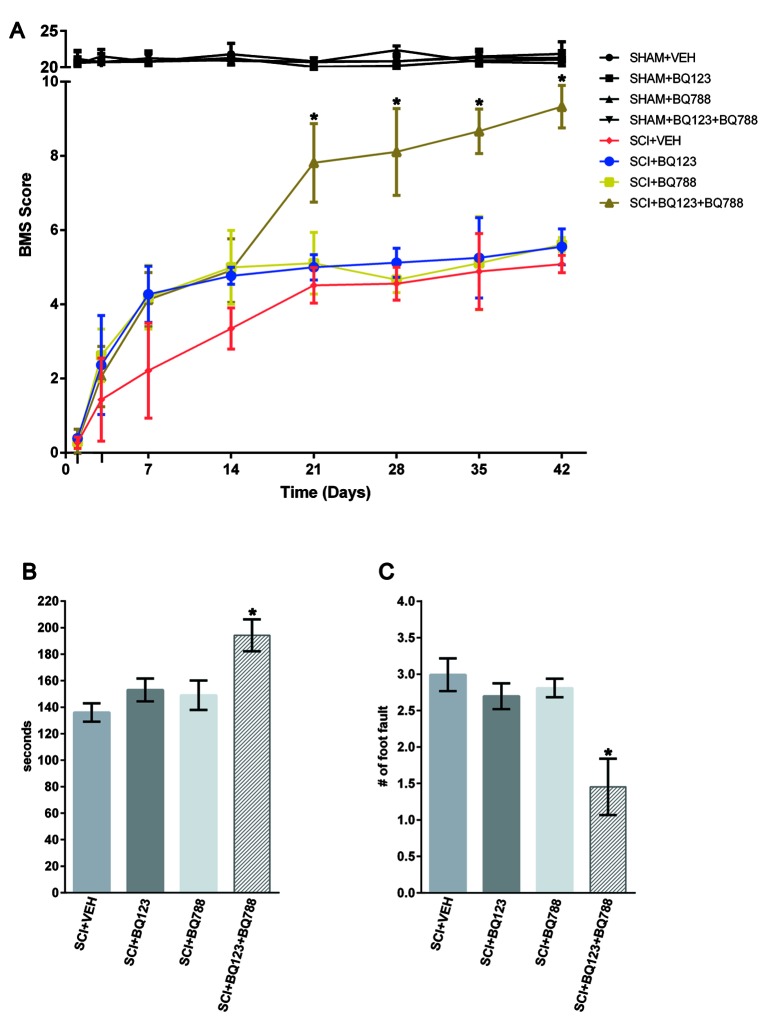
Treatment with BQ123 and/or BQ788 improves long-term functional recovery after spinal cord injury (SCI). Data are the mean ± standard error of the mean (SEM) of three independent experiments. ^*^P<0.05, SCI + BQ123 + BQ788 mice vs. SCI + vehicle control (VEH) mice (n=10/group). (A) Functional recovery as determined by the Basso Mouse Scale (BMS) in mice treated with BQ123 and/or BQ788. (B) Performance on a rotarod in BQ123- and/or BQ788-treated SCI mice. (C) Comparison of grid walking in BQ123 and/or BQ788 treated SCI mice.

To determine the effects of ETR blockade on the long-term functional recovery after SCI, locomotor performance was evaluated in the BMS open field test, on a rotarod, and on a grid-walking task for 6 weeks. Hind-limb movements were abolished immediately after SCI as assessed using the BMS scale (31). Although all the groups gradual improved in locomotor functional recovery after 3 days, this recovery was markedly higher between 21 and 42 days after SCI in the group with blockade of ETAR and ETBR (Fig. 5A). Subsequent assessment of the ability of SCI mice to maintain position on a rotarod and walk across a grid revealed similar functional improvements in SCI mice with blockade of ETAR and ETBR, relative to their respective controls (Fig. 5B–C).

## Discussion

ETR activation in the injured spinal cord triggers several pathologic responses, including leukocyte recruitment, superoxide generation, and BSCB breakdown, which exacerbate damage and impair neurological recovery (19–25). Although strategies to block ETRs in the injured spinal cord can promote locomotor function recovery, less is known about which ETR subtype influences neurological recovery processes after SCI (18,35,36). In this study, we have shown that blockade of ETAR and ETBR in SCI mice resulted in an early reduction in leukocyte infiltration, oxidative stress, and expression of inflammatory mediators and MMP-9. Notably, although all the SCI mice with blockade of ETAR or ETBR showed an early improvement in locomotor function, blockade of the two receptor types resulted in significant long-term locomotor function improvement. Collectively, these results provide evidence for the additive cooperation between ETAR and ETBR in influencing early secondary pathogenesis and long-term locomotor recovery after SCI.

BQ123 is a selective ETAR antagonist that has been used as a biochemical tool to investigate ETR function (37), whereas BQ788 is a selective ETBR antagonist used to demonstrate the role of ET-1 and ETBR in physiological and pathophysiological conditions (29). Results of previous studies have indicated that BQ123 and BQ788 may exert anti-inflammatory and anti-oxidative properties in patients with ischemic heart disease (27–29,38). In our experiments, the mice were treated with BQ123 and BQ788 pre- and post-SCI, which reduced ETAR and ETBR activity in the injured tissue, respectively. Thus, we assessed the specific roles of ETAR and ETBR in the injured spinal cord by using BQ123 and BQ788 in SCI mice.

Inflammatory neutrophils and monocytes are the first leukocytes to infiltrate the CNS after SCI (39–41). The number of circulating neutrophils increased 12–24 h post-injury (32,40), whereas the monocytes infiltrated into the CNS as early as day 1, reaching peak levels between 4 and 7 days after SCI (32). In our experiments, an increased number of circulating neutrophils and monocytes was detected in the peripheral blood in SCI mice. We also found that blockade of ETAR or ETBR pre- and post-SCI reduced circulating neutrophil numbers. Nevertheless, the blockade of ETBR or a combination of the two ETR subtypes decreased circulating monocytes in SCI mice. However, blockade of only ETAR had no effect on the number of monocytes after SCI. In parallel with this result, the expression levels of inflammatory mediators, such as TNF-α, IL-1β, IL-6 and iNOS, were upregulated after SCI, while the expression levels were significantly reduced following blockade of ETAR and ETBR. In addition, blockade of only ETBR did not decrease IL-1β and IL-6 expression levels in SCI mice. These results were consistent with previous findings (42–47) suggesting that the relationship between ETAR and ETBR in SCI mice is cooperative, and not antagonistic. The specific blockade of the two ETRs may inhibit inflammatory responses by suppressing the expression of inflammatory chemokines and reducing leukocyte infiltration following SCI-induced inflammatory mediator production.

Oxidative stress plays a critical role in secondary pathogenesis following SCI as it can lead to inflammation and apoptotic cell death. Increased oxidative stress reflects an imbalance between ROS and anti-oxidant levels (17,32). We assessed three oxidative stress parameters in SCI mice: HO-1, MDA and SOD. MDA is a reactive aldehyde resulting from the degradation of polyunsaturated lipids that causes toxic stress in cells (7). However, endogenous anti-oxidative enzymes SOD and HO-1 catalyze superoxide into oxygen and hydrogen peroxide (7,8,33,48). Consistent with previous observations (7,8,33,48), SCI induced an increase in MDA and reduced HO-1 and SOD expression. To the best of our knowledge, the results have shown for the first time that blockade of ETAR and ETBR in SCI mice significantly reversed reduction in MDA and SOD levels, thereby restoring the oxidative stress balance.

HO-1 is regarded as a sensitive and reliable indicator of cellular oxidative stress (7,8). Increased HO-1 expression in SCI rodent models can reduce neural tissue damage and improve locomotor function (32,49). HO-1 induction is regulated by the stress-responsive element and the Maf recognition element. Furthermore, HO-1 is rapidly induced by the specific regulation of oxidant-responsive transcription factors AP-1, NF-κB, and Nrf2/Keap1-Bach1 in oxidative stress (49,50). We found that blockade of ETBR or the two ETR subtypes resulted in a significant elevation of HO-1 expression in SCI mice, whereas the blockade of only ETAR had no effect. These results may be due to the critical role of ETBR on the ERK pathway signaling, which may affect the Nrf2/Keap1-Bach1 equilibrium, resulting in a decreased HO-1 expression (50,51).

Pro- and active MMP-9 is markedly accumulated in acute SCI, and it is able to degrade components of the extracellular matrix, such as microvasculature basal lamina and myelin basic protein (26,32). MMP-9 is derived primarily from neutrophils, although it can also originate from monocytes. It is likely that MMP-9 is released in response to activation of these cells following SCI. MMP-9 has been shown to contribute to early BSCB disruption, leukocyte infiltration, and white matter damage in SCI rodents (26,32). Furthermore, MMP-9-deficient mice show functional improvement in locomotion following cerebral focal ischemia (52). Similar to previous studies (30), we detected an increased MMP-9 expression and activity in SCI mice. We found that blockade of ETAR, ETBR, or a combination thereof significantly reduced levels of pro- and active forms of MMP-9. These results are highly dependent on the blockade of ETRs, which reduces leukocyte infiltration after SCI.

It was previously reported that use of pharmacological or genetic strategies to block ET signaling enhanced the recovery of locomotor function in SCI rodents (23,25). In our experiments, although blockade of only ETAR or ETBR in SCI mice gradually enhanced locomotor function recovery, improved long-term locomotor recovery was limited to SCI mice with blockade of ETAR and ETBR. These beneficial effects may be partially due to improvement of the SCI molecular environment, including reductions in inflammation, oxidative stress, and MMP-9 activation, all of which are dependent on cooperativity between the two ETR subtypes (26,32,48). It is also possible that ETAR or ETBR exert distinct effects on pro-inflammatory and oxidative stress states in SCI, which may subsequently influence neurological functional recovery. For example, blockade of vascular ETAR and ETBR and astrocyte ETBR after SCI may reduce ischemia and astrogliosis and facilitate neuronal survival, regeneration, and neurological function recovery (23). Further studies are needed to address the involvement of specific ETR subtypes in SCI.

Taken together, to the best of our knowledge, findings of this study provide the first evidence for collaboration between ETAR and ETBR in creating an environment that is hostile to neurological recovery in SCI. These results may serve as a foundation for developing combination ET-based therapy against multiple targets that alleviate early inflammatory response and oxidative stress and promote long-term functional recovery following SCI. Although the mechanisms involved in ETR blockade protection against SCI in mice requires further investigation, our data suggest that ETR blockade is an effective treatment for SCI and may be used to improve SCI recovery in humans in the future.
